# Patterns of complementary and alternative medicine use amongst outpatients in Tokyo, Japan

**DOI:** 10.1186/1472-6882-8-14

**Published:** 2008-04-23

**Authors:** Satoshi Hori, Iordan Mihaylov, Joana C Vasconcelos, Malcolm McCoubrie

**Affiliations:** 1 Department of Urology, West Suffolk Hospital, Bury St. Edmunds, UK; 2 Departments of Community and Mental Health, St George's, University of London, UK; 3 Centre for Applied Medical Statistics, Department of Public Health and Primary Care, University of Cambridge, UK

## Abstract

**Background:**

The use of complementary and alternative medicine (CAM) has been increasing rapidly throughout the world during the past decade. The use of CAM in the general Japanese population has been previously reported to be as high as 76%. This study aims to investigate the patterns of CAM use, perceived effectiveness and disclosure of CAM use to orthodox medical practitioners amongst patients attending typical primary and secondary care clinics in a busy district general hospital in Tokyo, Japan.

**Methods:**

The authors analysed data collected during March 2002 on patients attending general outpatient clinics held at Shiseikai Daini Hospital in Tokyo, Japan. Data was collected by use of self-completed questionnaires distributed to patients in the outpatient clinics waiting area. Statistical analysis was performed using chi-square tests of independence.

**Results:**

515 adults were approached to participate in this study and the overall response rate was 96% (n = 496). 50% of the patients were using or have used at least 1 CAM therapy within the last 12 months. The 5 most commonly used therapies were massage (n = 106, 43%), vitamins (n = 85, 35%), health foods including dietary supplements (n = 56, 23%), acupressure (n = 51, 21%) and kampo (n = 46, 19%). The majority of CAM users (75%, n = 145) found their CAM treatment to be effective (95% CI = 68–81%). Patients who were more likely to use CAM were females (p = 0.003) and those with a high number of medical conditions (p = < 0.0001). Only a small proportion of patients reported their CAM use to their physician (42%, n = 74). There was no significant difference in CAM use for the different age groups (p = 0.85), education level (p = 0.30) and financial status (p = 0.82).

**Conclusion:**

Patterns of CAM usage in the sample surveyed was high (50%). Despite this high prevalence rate and presumed acceptance of CAM in Japan, the reporting of CAM use by patients to their physicians was low (42%). It is therefore important that physicians are aware of the possibility that their patients may be using CAM and also increase their knowledge and understanding of these treatments.

## Background

### Definition of CAM

The US National Centre for Complementary and Alternative Medicine (NCCAM) defines complementary and alternative medicine as a 'group of diverse medical and healthcare systems, practices and products that are not presently considered to be part of conventional medicine' [1]. CAM therapies can be classified broadly into five main categories (table 1).

**Table 1:** NCCAM Classification of CAM therapies

1. Whole medical systems	Homeopathic medicine, naturopathic medicine, chiropractic, traditional Chinese medicine, Ayurveda etc.
2. Mind-body interventions	Meditation, prayer, mental healing, art, dance, music therapy etc.
3. Biologically based therapies	Herbs, vitamins, dietary supplements, Health foods, aromatherapy etc.
4. Manipulative and body based methods	Chiropractic or osteopathic manipulation, massage etc.
5. Energy therapies (Biofield therapies and bioelectomagnetic based therapies)	Reiki, Qi gong, therapeutic touch, electromagnetic fields etc.

### Use of CAM in Japan and around the world

The use of CAM around the world has increased dramatically in recent times and is still on the increase [2-4]. The reported CAM usage in western countries such as Australia, Canada, Denmark, Norway, USA and UK ranges from 9% to 69% [2,3,5-10].

The use of CAM in the Far East is presumed to be higher than those of the West, as many of the well known CAM therapies originated from this region. The exact prevalence rates of CAM usage in individual far eastern countries is not well known as there have been substantially fewer well conducted population weighted surveys investigating this. It has been reported that 60% of people in Taiwan use traditional Chinese medicine, a form of CAM [11]. In Japan, 76% of the general population was found to be using some form of CAM [12].

The exact definition of what constitutes CAM however is culturally dependent. In India for example, arurveda is practiced on a nationwide scale and is commonly regarded as orthodox medicine. In China, traditional Chinese medicine such as herbal medicine, acupuncture, acupressure, qi gond and t'ai chi chu'an are widely practiced alongside modern western medicine. In Japan, kampo (traditional Japanese herbal medicine) and acupuncture are officially considered a CAM therapy but part of these costs are covered by the Japanese public health insurance. Some Japanese practitioners of kampo and acupuncture would therefore object to their inclusion in CAM and would regard themselves as belonging to the authentic traditional medicine [13]. Thus, the definition of CAM differs substantially from country to country. We have classified kampo and acupuncture as CAM in this study to allow comparison with other studies in international literature.

With increasing use of CAM worldwide, orthodox medical practitioners need to be aware of the potential use of CAM by patients. This is especially important in Japan as the general population already has a high baseline usage of CAM [12]. With increasing life expectancy and an ageing population, it is likely that a substantial number of Japanese patients presenting to orthodox medical practitioners will be using some form of CAM.

### The Japanese healthcare system

Japan has a universal healthcare system in which individuals are covered either by the National Health Insurance scheme (for the self employed) or social insurance (for employees). Beneficiaries have to make some co-payments which are capped depending on income [14]. Provision of primary care in Japan is blurred with patients having the choice of visiting a community based primary care physician or a hospital outpatient clinic for assessment directly by a specialist without being charged a premium fee [15].

There have been a number of Japanese studies looking at the use of CAM in general practice [16,17] or in specialist outpatient clinics such as oncology [18-20] but we are unaware of studies looking at the prevalence of CAM in patients presenting to a typical district general hospital (DGH) outpatient clinic offering both primary and secondary care services. In this study, we document the prevalence, user characteristics, reasons for use, perceived effectiveness and disclosure of CAM use to orthodox medical practitioners in patients attending the primary and secondary care clinics in a busy Tokyo hospital.

## Methods

### Study population

The study population consisted of patients attending the general outpatient clinics held at Shiseikai Daini Hospital in Tokyo, Japan. The study was conducted between 21/03/2002 to 29/03/2002. Shiseikai Daini Hospital is a busy 300 bed Tokyo Women's Medical University affiliated district general hospital located in Setagaya-ku in Tokyo, Japan. Tokyo consists of 23 municipalities (special wards) that make up the metropolis. Setagaya-ku is the largest special ward within Tokyo with a population of 820,652 people in 2001 (Japan census 2000).

### Definition of CAM

We defined CAM using the US National Center for Complementary and Alternative Medicine (NCCAM) definition as a 'group of diverse medical and healthcare systems, practices and products that are not presently considered to be part of conventional medicine'. This definition was consistently used when verbal explanation of CAM was requested by patients.

The CAM categories included in our questionnaire were chosen to be comparable with previous CAM studies [4,21]. Our questionnaire was further adapted to include CAM categories that we felt would be important in the Japanese setting [22-24].

### Conduct of study

A two page semi-structured questionnaire consisting of 11 questions was initially developed in English. The questionnaire was then translated into Japanese by S.H. Parts of the questionnaire involved open-ended questions and all responses were analysed by S.H. The reliability and accuracy of the translation was tested by employing the back-translation method.

The draft Japanese questionnaire was piloted on 15 patients attending the general medical outpatient clinic at Shiseikai Daini Hospital on 20/03/2002 and some minor alterations were made prior to formally undertaking the survey. The two native Japanese speakers (SH and AH) distributing the questionnaires were calibrated prior to commencement of the study to ensure consistency in data recording and CAM definitions.

The questionnaire took on average 5 minutes to complete and included demographic data (age, sex, highest education level and financial status), current and past use of various CAM therapies within the last 12 months, current and past medical conditions suffered (within the last 3 years), reasons for using CAM therapies and its perceived efficacy and disclosure of CAM therapy use to their orthodox medical practitioner. Survey methods were approved by the research and ethics review board of Shiseikai Daini Hospital and strict confidentiality protocol was implemented and adhered to throughout the study.

Questionnaires were distributed twice daily to patients attending the adult general outpatient clinics run by the hospital and included the following specialties: obstetrics & gynaecology, neurology, neurosurgery, orthopaedics, rheumatology, cardiology, gastroenterology, ear, nose & throat, internal medicine, general surgery and dermatology. Specialist clinics such as oncology and pain clinics were not included in our survey. No specialty was canvassed more than twice daily. By covering all available non-specialist clinics and surveying these an equal number of times, we believe that we sampled a broad cross-section of patients attending these clinics.

The two native Japanese speakers (SH and AH) were in clear view of the respondents at all times to provide assistance upon request. In total, 515 adult patients were approached to participate and the overall response rate was 96% (n = 496). The exclusion criteria for our study were patients' age (less than 18), patients attending specialist (tertiary) clinics and those who were unable to understand written or verbal instructions to complete the questionnaire. The age cutoff of 18 was chosen as this study was only analysing the adult population.

### Statistical analyses

Univariate statistical analysis was performed using chi-square tests of independence (χ^2 ^test). A multi-variate analysis was also performed but this did not reveal any significant difference from the univariate analysis (data not shown). 95% confidence intervals were determined and a p-value of < 0.05 was considered statistically significant.

## Results

515 adults were approached to participate in this study and the overall response rate was 96% (n = 496). Of the 496 patients included in this study, 38% (n = 190) were male and 62% (n = 306) were female with age ranging from 18 to 92 (Table 2).

**Table 2:** Demographics of the population surveyed

**Characteristic**	**Variable**	**Sample** (n = 496)		**Users of CAM**^1^ (n = 246)		**p-value **(χ^2 ^test)
			
		**n =**	**%**	**n =**	**%**	
Gender	Male	190	38	78	32	0.003
	Female	306	62	168	68	
Age	< 20	4	1	2	1	0.85*
	20–29	58	12	26	11	
	30–39	68	14	38	15	
	40–49	53	11	29	12	
	50–59	85	17	40	16	
	60–69	109	22	51	21	
	70–79	83	17	42	17	
	80+	36	7	18	7	
Highest education†	University degree	155	31	77	31	0.30*
	Trade qualification	109	22	56	23	
	Secondary school	209	42	94	38	
	None (below secondary school)	13	3	7	3	
Financial status§	Not well off	67	14	33	13	0.82*
	Average	394	79	191	78	
	Well off	30	6	15	6	

* Linear-by-linear Association Chi-squared test

^1^CAM use within the last 12 months

† 10 patients (2%) did not answer this question

§6 patients (1%) did not answer this question

**Figure 1:**
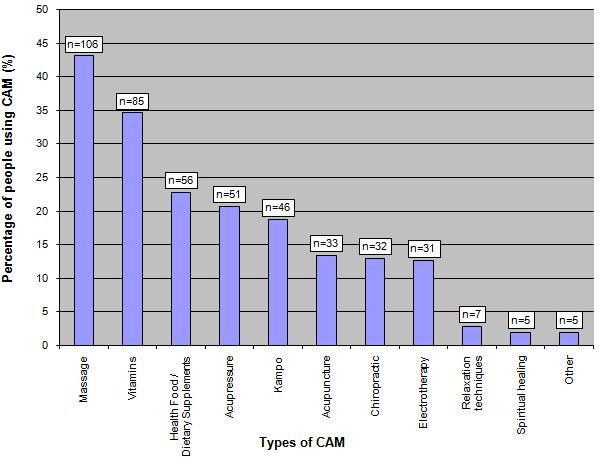
Types of CAM used by patients during the past 12 months.

50% (n = 246; 95% C.I 45% to 54%) out of 496 patients questioned were using or have used at least 1 CAM therapy within the past 12 months (Table 3). The 5 most common CAM therapies used amongst the 246 patients were massage (n = 106, 43%), vitamins (n = 85, 35%), health food including dietary supplements (n = 56, 23%), acupressure (n = 51, 21%) and kampo (n = 46, 19%) – see figure 1.

**Table 3:** Number of different CAMs used by individuals

Number of different CAMs used*	**Number (%) of people**†
1	116 (47%)
2	84 (34%)
3	24 (10%)
4+	22 (9%)

*within the last 12 months

†Total CAM users: 246

### Patterns and Reasons of CAM use

**Figure 2:**
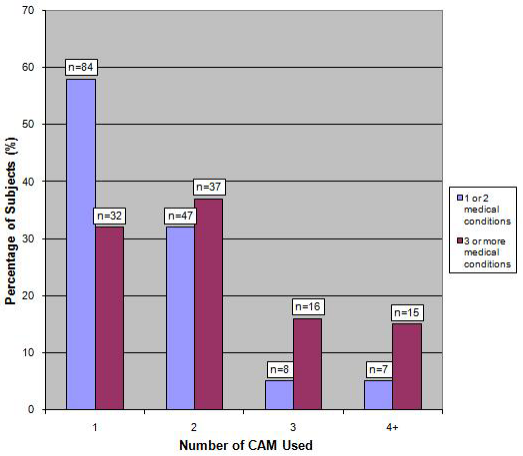
Patterns of CAM usage according to the number of medical conditions suffered.

In our sampled population, women (n = 168, 55%) used more CAM than men (n = 78, 41%) (p = 0.003). Patients suffering from 3 or more medical conditions were more likely to use a higher number of CAM than those suffering from less than 3 medical conditions – see figure 2 (p = < 0.0001). There was no significant difference in CAM use amongst different age groups (p = 0.85), in patients with higher educational level (p = 0.30) and in patients who are financially better off (p = 0.82) – see table 2.

The majority of patients used CAM for a specific medical reason. The 3 commonest medical conditions for which CAM was used were musculo-skeletal (n = 94, 38%), gastro-intestinal (n = 79, 32%) and cardiac problems (n = 76, 31%). The 5 commonest CAM used were massage (n = 106, 43%), vitamins (n = 85, 35%), health food including dietary supplements (n = 56, 23%), acupressure (n = 51, 21%) and kampo (n = 46, 19%). The commonest reasons given for using these CAMs are shown in table 4.

**Table 4:** Commonest reasons given for using the 5 most popular CAMs

**Type of CAM**	**Specific reasons stated for using CAM**	**% (n)**	95% C.I*
Massage (n = 106)	Musculo-skeletal problems	47% (50)	37% to 57%
	Recommended by friend	7% (7)	3% to 13%
	Neurological problems	5% (5)	2% to 11%
			
Vitamins (n = 85)	Musculo-skeletal problems	19% (16)	11% to 29%
	General wellbeing	14% (12)	8% to 23%
	To get better	9% (8)	4% to 18%
			
Health food/dietary supplement (n = 56)	Musculo-skeletal problems	21% (12)	12% to 34%
	General wellbeing	16% (9)	8% to 28%
	To get better	13% (7)	5% to 24%
			
Acupressure (n = 51)	Musculo-skeletal problems	35% (18)	22% to 50%
	Recommended by friend	10% (5)	3% to 21%
	General wellbeing	6% (3)	1% to 16%
			
Kampo (n = 46)	Musculo-skeletal problems	15% (7)	6% to 29%
	Gynaecological problems	13% (6)	5% to 26%
	Lack of perceived side effects	11% (5)	4% to 24%

*C.I = Confidence Interval

### Perceived effectiveness and reporting of CAM use to their orthodox practitioner

Out of the 246 patients who reported to have used at least 1 CAM during the past 12 months, 176 patients answered this question. Of these, only 74 patients (42%) informed their orthodox medical practitioner about their use of CAM. 102 patients did not report their use of CAM to their physician (58%). The majority of CAM users (75%, n = 145 out of 194) found their CAM treatment to be effective (95% CI 68%-81%) (p = 0.005).

## Discussion

This study investigated the use of CAM amongst a Japanese population attending the general outpatient clinics held at Shiseikai Daini Hospital in Tokyo, Japan. Half of those questioned (50%) were using or have used at least 1 CAM therapy within the last 12 months.

The main aim of this study was to investigate whether or not the usage of CAM in patients attending a DGH general outpatient clinics differs from that of the general population but interestingly we found that the top 5 CAM used in our population were also the same as those used in the general Japanese population as reported by Yamashita et al, 2002 [12].

We found that female patients were more likely to use CAM than males (p = 0.003), and this is consistent with previous published findings [4,25-28]. We also found that patients with a high number of medical conditions tend to use more CAM than others (p = < 0.0001). One explanation for this is that patients with more medical conditions will probably have had less success in treating their own health problems and their continued suffering may have prompted them to seek alternatives. Other studies have also shown that patients suffering from chronic diseases have a higher usage of CAM than those who don't [4,29-34].

In the present study, musculo-skeletal problems emerged as the top reason for patients using CAM (29%, n = 71). Musculo-skeletal problems and pain are frequently inadequately treated by orthodox medical practitioners and thus becomes troubling and chronic for many patients, who are therefore more likely to use CAM [29,30,33-38]. Dissatisfaction with conventional orthodox medicine was not a great determinant of patients resorting to using CAM, with only 1% (n = 6) of patients quoting this as a reason [39].

We found that most patients found their CAM to be of help (p = 0.005). Despite this, only 42% (n = 74) of the patients who responded to this question reported their use of CAM to their orthodox medical practitioner. This seemingly low percentage figure is in agreement with other studies [4,5,35,40,41]. It is somewhat surprising that with a high prevalence of CAM usage and presumed acceptance of CAM therapies in Japan, reporting of CAM usage by individuals to their orthodox medical practitioners was low. Our sample population consisted of those patients who were attending and seeking help from orthodox practitioners. Patients therefore may have found it hard to report their CAM use for fear of anticipating a negative response and disapproval from their doctor [35].

### Limitations of the study

This study analysed a population of patients attending a typical busy Tokyo district general hospital outpatient department and is therefore not representative of the general population. By approaching consecutive outpatient presentations and systematically sampling the different specialities, the potential for sampling bias was minimised. Patients were asked to fill a questionnaire and to recall the use of CAM in the past year therefore they may have been subjected to recall bias.

Although our prevalence rate was high (50%), it is lower than those reported by Yamashita et al in 2002 [12]. They reported a prevalence rate of 76%. There are a number of reasons why their value might be higher than that found in this study. Firstly, Yamashita et al sampled a nationwide, population weighed sample which is likely to be more representative of the general population compared to our study. Although we tried to minimise sampling bias with our methodology, people who attend hospital outpatient clinics are already a self selected group of patients who are attending these clinics for a specific medical reason. Thus the usage and reasons for CAM use in our patient group is different from that of the general population.

Secondly, the definition of CAM used in this study differed from that used by Yamashita et al (2002) and this may account for the difference in the prevalence rate. However, in order to allow our results to be compared to other studies in the international literature, the CAM categories included in our questionnaire were chosen to be comparable with previous CAM studies [4,21]. We further included CAM categories that we felt would be important in the Japanese setting [22-24].

Unlike previously reported [25], we have not found higher education level to be associated with a higher usage of CAM (p = 0.30). One of the reasons why we may not have found a significant difference in education being associated with CAM use may be because only 3% (n = 13) of our responders left education at secondary school level and below. This figure is considerably lower than that of the Japan census (2000) where 26.6% of the Japanese population was reported to have an education level of secondary school level and below. An explanation is that those who attend hospital outpatient clinics are patients who are more health conscientious and therefore more likely to have a higher level of education [42].

Financial status also failed to reveal any significant difference (p = 0.82), contrary to those reported by Wiles and Rosenberg [25]. Japan, unlike many other countries, lacks the distinct boundaries that exist between different social classes. Significant number of Japanese people are middle class or above and this may explain in part the reason why financial status did not predict the usage of CAM. Another reason may be the way in which financial status was analysed in this study. We asked patients whether they thought they were "not well off, average or well off". Patients may be reluctant to admit their real financial status and thus may write down "average" as a default answer. A better method to survey this would have been to ask patients to reveal their income; however this might have made people more reluctant to participate in this study and thus decrease the overall completion rate.

A number of questions within the survey were noted to have a lower response rate than others. Questions relating to highest educational level, financial status and CAM use reporting to their orthodox medical practitioner were noted to be such cases. It is reasonable to expect this, as many people do not wish to disclose this more intimate data. Nevertheless, we felt that we had enough respondents to justify these results being included in our analysis.

### Applicability of the study

Despite these limitations, there are a number of important findings in this study. We have found that a high proportion of Japanese patients who attend hospital outpatient clinics use CAM and do not disclose this fact to their doctors. This is important in the Japanese setting where provision of primary care in Japan is blurred with patients having the choice of visiting a community based primary care physician or a hospital outpatient clinic for assessment directly by a specialist [15]. Orthodox practitioners should be aware that their patients may be using CAM and should routinely enquire about this during consultation.

Although CAM is often seen by patients as the "healthy alternative with low or no side effect profile", numerous papers have highlighted the possible side effects of certain CAM therapies. There is a risk of tissue trauma and infection from acupuncture and carcinogenic potential of some aromatherapy oils. The herbs St John's wort and Gingko biloba carry a risk of bleeding and risk of interactions with many drugs. Reports of mechanical injury following acupuncture leading to pneumothorax, cardiac tamponade and spinal cord injury have all been reported [21,43-51]. With increasing CAM popularity, it is important that orthodox medical practitioners are aware of these potential side effects posed by the usage of CAM and should routinely enquire patients regarding CAM use.

## Conclusion

This present study showed that 50% of patients surveyed were currently using or have used at least 1 CAM therapy within the last 12 months. Socio-demographic variables did not predict use, and reported use of CAM to orthodox practitioners was low and perceived effectiveness was high. It is of paramount importance therefore that doctors increase their understanding and awareness of potential CAM usage by their patients.

## Competing interests

The authors declare that they have no competing interests.

## Authors' contributions

SH came up with the original idea. SH and IM contributed equally in obtaining the completed questionnaires and analysing the data. JCV double checked the results and performed the statistical analysis. MM participated in the design and coordination of the study and helped to draft the manuscript. All authors read and approved the final manuscript.

## Pre-publication history

The pre-publication history for this paper can be accessed here:


